# Effect of Quantitative Structural Properties and Drug Formulation in Four Cannabinoids (Cannabidiol, Cannabigerol, Cannabichromene, and Cannabinol) on Their Lymphatic Transport after Enteral Administration in Rats

**DOI:** 10.1021/acs.molpharmaceut.4c01357

**Published:** 2025-07-04

**Authors:** Pavel Ryšánek, Petr Jelínek, Hynek Housar, Petr Kozlík, Tomáš Křížek, Anežka Nováková, Michaela Sklenárová, Viktória Paulusová, Sara Merdita, Mahak Arora, Olesia Symkanych, Monika Šteigerová, Eliška Zmeškalová, Ondřej Slanař, Miroslav Šoóš, Martin Šíma

**Affiliations:** a Institute of Pharmacology, 112302 First Faculty of Medicine and General University Hospital in Prague, Charles University, Albertov 4, 128 00 Prague, Czech Republic; b Department of Chemical Engineering, Faculty of Chemical Engineering, University of Chemistry and Technology, Technická 3, 166 28 Prague, Czech Republic; c Department of Analytical Chemistry, Faculty of Science, Charles University, Hlavova 2030/8, 128 43, Prague, Czech Republic; d Institute of Physics of the Czech Academy of Sciences, Na Slovance 2, 182 00 Prague, Czech Republic

**Keywords:** cannabinoids, pharmacokinetics, oral bioavailability, lymphatic transport, lymph-targeting, nanoemulsion, quantitative structure−activity relationship (QSAR)

## Abstract

The effect of quantitative structural properties of drugs on the extent of lymphatic transport is not well understood. Our study aimed to describe these principles in four cannabinoids, cannabidiol (CBD), cannabigerol (CBG), cannabichromene (CBC), and cannabinol (CBN) administered as oil solutions and nanoemulsions. A series of studies in jugular vein cannulated rats and anesthetized mesenteric lymph duct cannulated rats was conducted to measure drug oral bioavailability and lymphatic transport. Log P was measured, and quantitative structural properties were correlated to the extent of lymphatic absorption. Nanoemulsion did not increase the absolute bioavailability via lymph in CBD but led to an 8-fold increase in CBG and a 3-fold increase in CBC and CBN. There was an even higher increase in the absolute bioavailability via portal vein (11-fold for CBD, 71-fold for CBG, 8-fold for CBC, and 13-fold for CBN). Relative bioavailability via lymph increased with decreasing smallest orthogonal molecular size and topological polar surface area. Nanoemulsion did not affect the total oral bioavailability but led to an increased absorption into portal blood. Intestinal lymphatic transport plays a major role in the absorption of CBD, CBG, CBC, and CBN. Planarity of the molecule and low surface polarity could be crucial structural features facilitating lymphatic transport.

## Graphical abstract

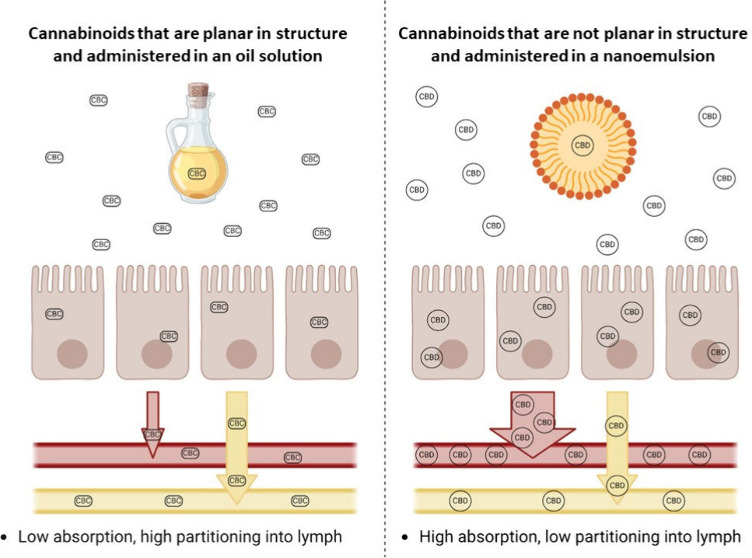

## 1 Introduction

Lymphatic transport of drugs after oral administration plays an important role in the absorption of highly lipophilic compounds with log P > 5.^1–4^ After solubilization in the gastrointestinal tract, these compounds are absorbed into enterocytes, the intestinal epithelial cells, where they are incorporated into chylomicrons. Chylomicrons are large lipoprotein particles produced specifically in the intestines that are naturally involved in the transport of highly lipophilic molecules. Their size/diameter is too large to pass into the intestinal blood capillaries. Therefore, they take an alternative route via the intestinal lymphatic system. Chylomicrons enter the lacteals (lymph capillaries) and are transported through a network of lymphatic vessels and mesenteric lymph nodes until they reach the thoracic duct, where intestinal lymph mixes with lymph from the lower parts of the body. All the lymph eventually enters the systemic blood circulation at the confluence of the thoracic duct with the jugular and subclavian veins.

The transport through the intestinal lymphatic system can have a major impact on the drug pharmacokinetics. It can increase the absolute oral bioavailability because the lymph represents an additional gateway into the systemic circulation besides the standard transport through the portal vein. Another mechanism for increasing oral bioavailability is by avoiding first-pass metabolism in the liver. Mesenteric lymph is a dominant (sometimes even exclusive) source of systemically available drug for compounds with a high extraction ratio in the liver.^5,6^ Lymphatic transport can improve the efficacy of drugs that act against components of the lymphatic system, such as immunosuppressants, anti-HIV and anticancer drugs by reaching high exposure in the mesenteric lymphatic system.^7,8^

Given the possible advantages of targeting drugs into the mesenteric lymph, various approaches have been tested to increase lymphatic transport. However, summarizing all the available literature on in vivo lymphatic transport data, it seems that different strategies must be implemented based on the drug’s lipophilicity. In highly lipophilic compounds (log P > 5), it may be sufficient to use a lipid-based drug formulation (oil solution, o/w emulsion, self-nanoemulsifying system, etc.), because lipids (especially long-chain triglycerides) promote the physiological assembly of chylomicrons in which the drugs are dissolved and transported through the lymph.^9,10^ Targeting drugs with lower lipophilicity (log P < 5) into the mesenteric lymph is much more challenging. The only functional technique discovered so far is a synthesis of a lipophilic prodrug by covalently binding a lipid residue to the original drug molecule. Such complex molecule has log P > 5 and is effectively transported into the lymph. Examples of drugs successfully targeted into the lymph despite their insufficient lipophilicity are e.g. mycophenolate, valproate, and paracetamol.^11–13^ There are few reports on targeting low-lipophilicity drugs into the lymph without lipophilic prodrug synthesis. However, the extent of the lymphatic transport for these compounds has been investigated using a noninvasive lymphatic transport measurement, which has been shown to significantly overestimate the results.^14–16^

Cannabinoids are an important group of compounds found naturally in cannabis or produced synthetically and frequently used as registered medicines, nutritional supplements or abused as recreational drugs. They are lipophilic, mostly with log P > 5, and are therefore suitable model drugs for lymphatic transport driven absorption. The most frequently investigated cannabinoids are THC (tetrahydrocannabinol), the main psychoactive compound in cannabis, and CBD (cannabidiol), the second most abundant but nonpsychoactive cannabinoid. Besides these two, there is a variety of “minor” cannabinoids with much less pharmacological data available. In this work, we focus on four nonpsychoactive compounds: the “major” cannabinoid CBD, and three “minor” cannabinoids CBG (cannabigerol), CBC (cannabichromene), and CBN (cannabinol). Their structures are shown in Figure 1.

**Figure 1:**
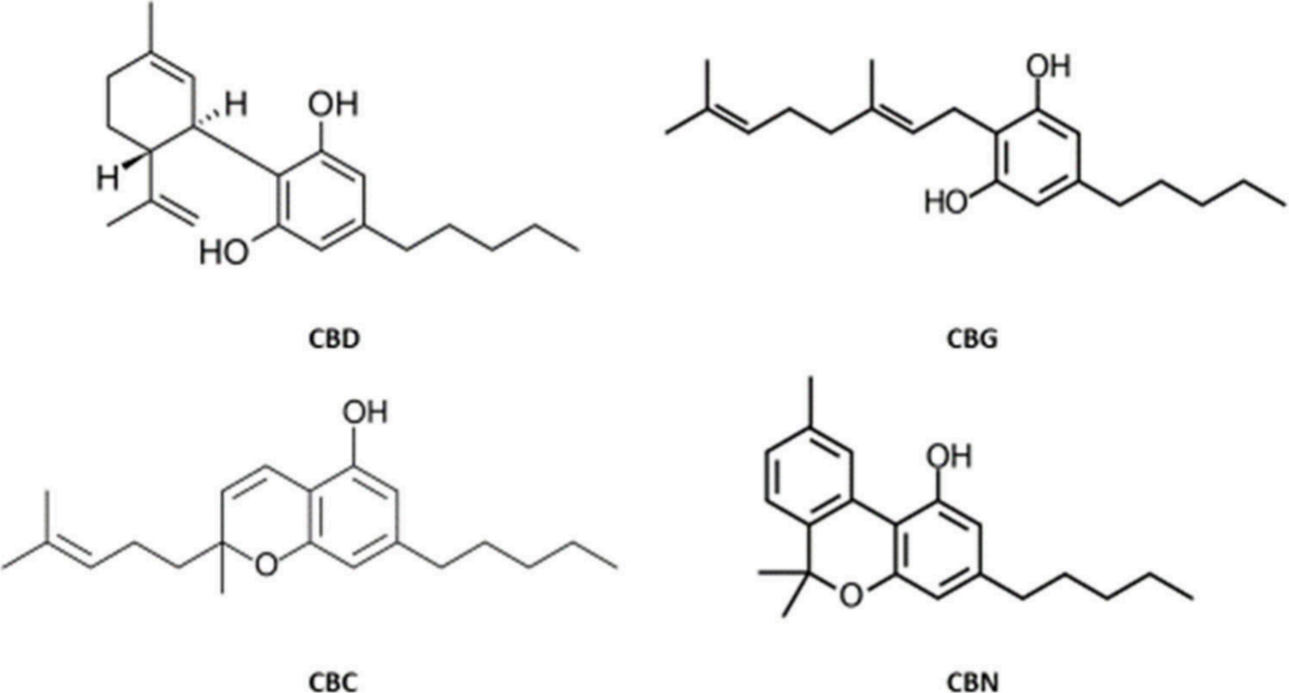
Structures of the four tested cannabinoids: cannabidiol (CBD), cannabigerol (CBG), cannabichromene (CBC), and cannabinol (CBN)

CBD has been registered for the treatment of specific forms of childhood epilepsy. It is administered orally in a formulation based on sesame oil solution (Epidyolex). Besides that, CBD, together with THC, are the active components of authorized cannabis extract registered for the treatment of spasticity in patients with multiple sclerosis (Sativex). CBD has a very limited oral bioavailability, estimated at <10% in man.^17^ When taken with food, the bioavailability increases up to 4-fold.^18^ Preclinical pharmacokinetic data are consistent with the human observations: CBD had a bioavailability of 8% and 22% in rats when administered in a lipid-free formulation and in a long-chain triglycerides-based formulation, respectively.^19^

CBG is a much less known cannabinoid compared to CBD. There are no registered drug products containing this compound worldwide. Nevertheless, it is marketed as a nutritional supplement and various pharmacological effects have been reported in preclinical studies. CBG possesses antioxidant, anti-inflammatory, and antitumoral activities, and has antianxiety, neuroprotective and appetite-stimulating effects.^20^ Absolute oral bioavailability is not known. When administered to dogs, CBG was rapidly absorbed with an average T_max_ of 0.75 h.^21^ When coadministered with food, the speed and extent of absorption did not change significantly.

CBC has shown an anti-inflammatory and antihypertensive effect in preclinical studies.^22,23^ After oral administration to man, it absorbs with moderate speed (T_max_ 2–4 h). Absolute oral bioavailability is not known.^24^

CBN was successfully tested in the treatment of dermatological disease epidermolysis bulosa.^25^ After oral administration to rats, it was absorbed with T_max_ of 1.5 to 3 h.^26^ Absolute oral bioavailability was not determined.

One way of increasing the bioavailability of lipophilic compounds with problematic dissolution in the gastrointestinal tract is the development of formulations where the active substance is dispersed in small lipid droplets (microemulsions, nanoemulsions, self-emulsifying systems etc.). This method was successfully used e.g. in ivacaftor, where the bioavailability was increased 7-fold in Beagle dogs and previously present positive food effect was eliminated.^27^ There are discussions, whether this bioavailability increase is solely due to better solubilization of the active substance in the gastrointestinal tract, or if there are some additional mechanisms like e.g. involvement of the intestinal lymphatic transport. Unfortunately, there are only very few studies comparing the lymphatic transport of one particular compound administered in a basic formulation (aqueous or oil solution) and in an advanced formulation containing a solubilized drug.^2^ In our recently published preliminary data, the CBD oral bioavailability did increase mainly due to increased nonlymphatic transport when administered as a microemulsion compared to a simple oil solution.^28^

There are specific physicochemical properties and molecular structure characteristics that help the drugs partition into the lymph after intestinal absorption. The role of log P has been postulated already several decades ago by Charman and Stella.^1^ The same authors also discussed the importance of compound solubility in long-chain triglycerides. However, at least one molecule was later found (penclomedine) with a very limited partitioning into lymph despite high lipophilicity (log P > 5) and high solubility in long-chain triglycerides (>50 mg/g).^29^ Moreover, log P was absent in the mathematical model developed by Holm and Hoest predicting precise lymphatic transport of drugs that was based on in vivo data from 19 molecules.^30^ Instead, factors such as the size of the hydrophilic area in the molecule and hydrophilic–lipophilic ratio did play a role in this model. It is therefore evident that no single physicochemical property or structural pattern can be used to precisely predict the extent of lymphatic transport and the result is formed by an interplay of more variables. Unfortunately, there is a lack of experimental data investigating molecular structure characteristics like molecular size, molecular shape, and surface polarity on the extent of lymphatic transport.

The aim of this study was to evaluate whether an advanced drug formulation (a nanoemulsion) increases bioavailability via portal blood or via lymphatic transport and to assess the importance of quantitative structural properties of the molecule for the extent of lymphatic transport. To achieve these aims, we investigated four similar compounds CBD, CBG, CBC and CBN and their quantitative structure–activity relationship (QSAR) with lymphatic transport after oral dosing in a simple formulation (oil solution) and a nanoemulsion.

## 2 Materials and Methods

### 2.1 Drug Formulations

In the pharmacokinetic and lymphatic transport studies, the cannabinoids CBD, CBG, CBC, CBN (purchased from Pharmabinoid, Netherlands) were administered in sunflower oil and nanoemulsion. The required amount of cannabinoid was dissolved in sunflower oil of pharmaceutical quality (Fagron, Czech Republic) to obtain a reference formulation. The composition of the nanoemulsion formulation was optimized. In brief, a three-component system was selected—propylene glycol monocaprylate (PGMC; Gattefosse’, France), Kolliphor EL (CR-EL; BASF, Germany) and the third component acting as a cosurfactant and solubilizer was subject to optimization. Diethylene glycol monoethyl ether (TRSC; Gattefosse’, France), ethyl alcohol – 99.8% (ETOH; Penta, Czech Republic) or propylene glycol – 99.5% (PRGL; Penta, Czech Republic) were tested as cosurfactant and solubilizer. According to the optimization results and literature research, TRSC was chosen for further *in vivo* experiments. Further information regarding optimization is shown in the Supporting Information. The nanoemulsion formulation used in the pharmacokinetic study for both the oral and the intravenous administration was prepared as follows. Cannabinoid was dissolved in the oil phase (50 wt % of CR-EL, 30 wt % of TRSC and 20 wt % of PGMC). Next, four parts of water with respect to one part of oil (by weight) were added dropwise to the oil mixture at mild stirring. The final formulation was kept out of light and stored in the fridge before application.

### 2.2 Animals

All animal experiments were performed with approval from the Ministry of Education, Youth and Sports, Czech Republic (MSMT-26838/2021-4). All efforts were made to minimize animal suffering. Male Wistar rats (weight 300–450 g, age 3–5 months) were purchased from Velaz s. r. o., Prague, Czech Republic. They were housed under standard conditions (12-h light/dark cycle, 22 °C temperature, and 50% humidity) in cages with wood shavings bedding (two rats per cage during acclimation, one rat per cage during experiment) and fed on water and granulated diet ad libitum. The acclimation period took at least 1 week. The animals were randomly assigned to experimental groups.

### 2.3 Pharmacokinetic Studies

Absolute oral bioavailability and other pharmacokinetic parameters were determined in a series of two-period, crossover studies, where the cannabinoids were administered intravenously in the first period, and orally in the second. Both jugular veins were cannulated (3 Fr polyurethane catheter, Instech Laboratories, Plymouth Meeting, USA). One catheter was used for intravenous dosing, and the second one was used for repetitive blood sampling in order to avoid sampling cannula contamination with highly concentrated intravenous drug solution. The anesthesia was performed using a rapid inhalational induction with isoflurane (IsoFlo, Zoetis, Czech Republic) followed by intramuscular injection of ketamine 100 mg/kg (Narkamon, Bioveta a.s., Czech Republic) and xylazine 5 mg/kg (Rometar, Bioveta a.s., Czech Republic). After a three-day recovery period, the rats were intravenously dosed with the particular cannabinoid (1 mg of CBD, 1.5 mg of the other three cannabinoids in the form of nanoemulsion, volume 100 μL). Systemic blood (100 μL) was drawn at 5, 15, and 30 min and at 1, 2, 4, 6, 8, 10, and 24 h(s) postdose. After a two-day wash-out period, the dosing via oral gavage followed (10 mg of CBD, 15 mg of the other three cannabinoids in the form of nanoemulsion, volume 1 mL). Systemic blood (100 μL) was drawn at 0 (predose), 0.5, 1, 2, 3, 4, 5, 6, 8, 10, and 24 h(s). Blood samples were centrifuged (4500 rpm for 10 min) and serum was extracted and stored in −80 °C until analysis. The laboratory was unaware of animal assignment to particular experimental groups (laboratory blinding).

### 2.4 Lymphatic Transport Studies

Anesthetized mesenteric lymph duct cannulated rat model was used for the lymphatic transport measurement as previously described with slight modifications.^31^ Briefly, rats were left on normal diet and given 1 mL of olive oil 1 h prior to the surgery to visualize the mesenteric lymph (milky white color). They were anesthetized with intramuscular xylazine 5 mg/kg and ketamine 100 mg/kg after a rapid isoflurane induction. A transverse laparotomy was performed. The mesenteric duct was identified cranially to superior mesenteric artery and cannulated with heparin prefilled 0.91 mm O.D., 0.46 mm I.D. polyethylene catheter (Instech Laboratories, Plymouth Meeting, USA). The catheter was fixed in place with two to three drops of tissue adhesive (Histoacryl, B. Braun Surgical, S.A., Spain). A duodenal catheter was also placed (polyethylene, 0.97 mm O.D., 0.58 mm I.D.) via a small duodenotomy and fixed with tissue adhesive. The abdominal wall was sutured in two layers, with both catheters leaving the abdominal cavity through the right flank. At the end of the procedure, the right jugular vein was cannulated for blood sampling.

The rats were then placed on heated pads and covered with blankets to prevent heat loss. Cannabinoids in oil solution or nanoemulsion were then dosed slowly via duodenal catheter over 30 min. The administered dose was 10 mg of CBD and 15 mg of CBG, CBC and CBN (volume 1 mL). Whole lymph was collected in regularly changed Eppendorf tubes from the time the dosing started. When the dosing was finished, continuous hydration with normal saline at a rate of 3 mL/h intraduodenally followed using an infusion pump (Perfusor compactplus, B. Braun Melsungen AG, Germany). Anesthesia was maintained throughout the rest of the experiment and additional ketamine intramuscular boluses were given whenever necessary. Eppendorf tubes were changed hourly and systemic blood was drawn at the same time points. Blood samples were centrifuged (4500 rpm for 10 min) and serum was extracted. Lymph volume was measured gravimetrically and the samples were further processed without additional adjustment. All samples were stored in −80 °C until analysis. Laboratory was unaware of animal assignment to the particular experimental groups (laboratory blinding).

### 2.5 Bioanalysis

The concentrations of the cannabinoids under study, namely CBD, CBG, CBC and CBN were determined in both serum and lymph samples. An ultrahigh performance liquid chromatography-tandem mass spectrometric (UHPLC-MS/MS) method was developed for the quantification of each cannabinoid, employing an isotopically labeled internal standard (IS). The samples underwent identical processing procedures for each cannabinoid: 80 μL of 100% acetonitrile containing 30 ng/mL of an appropriate internal standard was added to 20 μL of the sample. After vortexing and centrifugation at 10000 × g for 8 min, 60 μL of the supernatant was transferred into an LC vial. For the UHPLC-MS/MS analysis, the Shimadzu UHPLC Nexera X3 coupled with a Triple Quad 8045 tandem mass spectrometer (Shimadzu, Kyoto, Japan) was used. Chromatographic analysis was performed on a Poroshell 120 EC-C18 column (50 × 2.1 mm; 1.9 μm; Agilent Technologies, Inc., Santa Clara, CA, USA). The mobile phase consisted of 0.1% formic acid in deionized water (Solvent A) and methanol with 0.1% formic acid (Solvent B). The flow rate of the mobile phase was maintained at 0.4 mL/min, except for the CBC determination method, which had a flow rate of 0.45 mL/min. The injection volume was 2 μL. The temperature of the column was kept at 40 °C and samples were thermostated at 10 °C. The optimized gradient elution proceeded as follows for each cannabinoid: CBD (min/% B): 0/50, 0.5/50, 2.5/90, 3.5/90, 4.0/50, and 5.5/50; CBG (min/% B): 0/50, 0.5/50, 2.5/90, 4.0/90, 4.5/50, and 7.0/50; CBC and CBN (min/% B): 0/40, 0.5/40, 2.5/90, 4.0/90, 4.5/40, and 7.0/40. The MS/MS spectrometer was operated in a positive mode. The applied conditions of the electrospray ion source were: nebulizing gas flow: 3 L/min, heating gas flow: 10 L/min, interface temperature: 300 °C, desolvation line temperature: 250 °C, heat block temperature: 400 °C, and drying gas flow: 10 L/min. The MS/MS measurement was performed in multiple reaction-monitoring mode (MRM). MRM transitions of 315.2 > 193.1 (Q1 prebias −16 V, Q3 prebias −20 V and collision energy −22 V) and 318.2 > 196.1 (Q1 prebias −16 V, Q3 prebias −22 V and collision energy −35 V) were monitored for CBD and CBD-d3, respectively. MRM transitions of 315.5 > 193.2 (Q1 prebias −13 V, Q3 prebias −20 V and collision energy −20 V) and 324.4 > 202.2 (Q1 prebias −17 V, Q3 prebias −21 V and collision energy −20 V) were monitored for CBC and CBC-d9, respectively. MRM transitions of 317.2 > 193.2 (Q1 prebias −15 V, Q3 prebias −20 V and collision energy −19 V) and 326.3 > 202.2 (Q1 prebias −16 V, Q3 prebias −21 V and collision energy −18 V) were monitored for CBG and CBG-d9, respectively. MRM transitions of 311.3 > 223.2 (Q1 prebias −17 V, Q3 prebias −21 V and collision energy −21 V) and 314.3 > 223.2 (Q1 prebias −17 V, Q3 prebias −24 V and collision energy −22 V) were monitored for CBN and CBN-d3, respectively.

The calibration curves were constructed in each blank matrix (serum and lymph) with seven concentrations by plotting the ratio of the peak area of cannabinoid to that of IS against cannabinoid concentration. The weighted least-squares linear regression method was used with a weighting factor of 1/x2, which improved the accuracy in low concentrations. The methods were linear (coefficients of determination (R2) higher than 0.9995) in the concentration ranges of 1–1000 ng/mL for CBD, and 2.5–1250 ng/mL for CBG, CBC, and CBN. The methods were validated according to the requirements of the European Medicines Agency (EMA) Guideline on bioanalytical method validation in terms of linearity, lower limit of quantification (LLOQ), upper limit of quantification (ULOQ), accuracy, precision, selectivity, recovery, carry-over effect, matrix effects, robustness, dilution integrity, and stability of quality control (QC), and have been demonstrated to be suitable for their intended purpose.^32^ All validation parameters met the acceptance criteria defined in the EMA guideline.

### 2.6 Log P Measurement

There are only calculated log P values available for CBD, CBG, CBC and CBN (range 6.1–7.4). Therefore, log P was measured experimentally for all four cannabinoids during this study. The partition coefficient describes the distribution of a tested compound in a two-phase octanol–water system. It is defined as a ratio of concentrations of the un-ionized compound in octanol and water at equilibrium. Cannabinoids were weighed (2 mg) and dissolved in 2.5 mL of octanol, followed by the addition of 2.5 mL of deionized water. The mixture was shaken for 2 h and then allowed to separate for 24 h. Twenty μL of the aqueous (bottom) phase was transferred into an LC vial, diluted with 60 μL of an appropriate IS at a concentration of 30 ng/mL, and analyzed using the aforementioned UHPLC-MS/MS method. The experiment was repeated five times for each cannabinoid.

### 2.7 Quantitative Structure Determination

First, an optimal, energy-minimized conformation was obtained by molecular mechanics modeling in the software Avogadro, version 1.2.0.n (an open-source molecular builder and visualization tool). The force field GAFF, which is optimized for drug molecules, was used. The initial conformations of the cannabinoids were obtained either from available crystal structures (CBD and CBG) or generated using SMILES descriptors (CBC and CBN). The size of the molecules was calculated as the smallest, medium, and largest orthogonal dimensions in Å (10^–10^ m) using the software rPluto, version 5.26 (The Cambridge Crystallographic Data Centre, Cambridge, UK).

### 2.8 Data Analysis and Statistics

Serum and lymph concentrations in all studies were dose-normalized to 1 mg/kg prior to further calculations. AUC values were determined using linear trapezoidal rule. Exact actual sampling times were used for this purpose. Scheduled sampling times were used for mean concentration plotting in the graphs. PK-solver add-on for MS Excel was used for all basic pharmacokinetic calculations.^33^ GraphPad Prism version 10.2.2 (GraphPad Software, San Diego, CA, USA) was used for all statistical analyses and graph plotting. Unpaired Student’s *t* test was used to compare pharmacokinetic and lymphatic transport parameters between the experimental groups. Level of significance was set to *p* < 0.05.

### 2.9 Calculation of Lymphatic Transport Parameters

Lymphatic transport parameters were defined and calculated as previously described.^2,14,34^ Briefly, absolute bioavailability via lymph (F_AL_) was defined as percentage of administered drug dose absorbed into the lymph. It was determined directly from lymph volume and drug concentration in lymph duct cannulated rats. Absolute bioavailability via portal vein (F_AP_) was analogically defined as percentage of administered drug dose reaching the systemic circulation after direct absorption into blood. It was calculated using equation

1 $$F_{\mathrm{AP}}={\mathrm{AUC}}_{\mathrm{ent}}/{\mathrm{AUC}}_{\mathrm{iv}}$$

where AUC_ent_ is the area under the dose-normalized blood concentration–time curve after enteral dosing in lymph duct cannulated (i.e., lymph deprived) rats and AUC_iv_ is the respective parameter in a separate intravenously dosed group. Total absolute bioavailability (F) in lymph duct cannulated rats was calculated as a sum of F_AL_ and F_AP_. In normal animals with no lymph duct cannulation, F was calculated using standard formula for oral bioavailability:

2 $$F={\mathrm{AUC}}_{\mathrm{po}}/{\mathrm{AUC}}_{\mathrm{iv}}$$

Relative bioavailability via lymph (F_RL_) was defined as the percentage of systemically available drug that was absorbed via lymph. It was calculated using the equation

3 $$F_{\mathrm{RL}}=F_{\mathrm{AL}}/F$$

## 3 Results and Discussion

### 3.1 Pharmacokinetics

Pharmacokinetic profiles for all four cannabinoids after oral and intravenous administration are shown in Figure 2. The corresponding pharmacokinetic parameters are summarized in Table 1. The absolute oral bioavailability varied significantly from 4.4% in CBG nanoemulsion to as much as 52% in CBD oil solution. The bioavailability from the oil solution and nanoemulsion did not differ significantly in the particular cannabinoids with the exception of CBN, where the compound achieved a higher systemic exposure when administered in an oil solution. The drugs were absorbed at a low speed after oil solution administration (T_max_ of 4.9 to 6.8 h) and at a moderate speed after nanoemulsion administration (T_max_ of 2 to 3.5 h). The elimination was quite fast in CBD, CBG and CBC with *T*_1/2_ of 2 to 2.5 h after intravenous dosing, whereas the CBN elimination was slower with *T*_1/2_ of 5 h.

**Figure 2:**
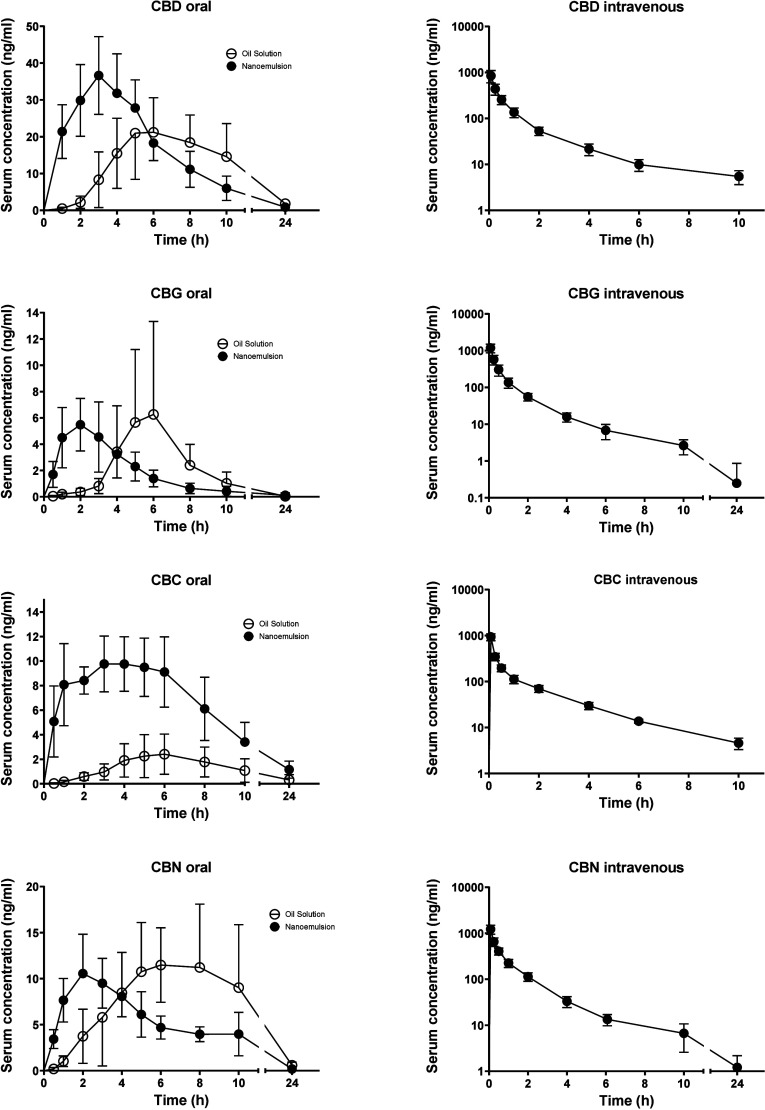
Mean ± SD serum pharmacokinetic profiles of CBD, CBG, CBC, and CBN after administration in the form of an oil solution (oral) and nanoemulsion (oral and intravenous) to rats (*n* = 5 to 8, see Table 1). The administered dose was 10 mg of CBD and 15 mg of the other cannabinoids in oral dosing (volume 1 mL) and 1 mg and 1.5 mg, respectively, in intravenous dosing (volume 0.1 mL). All concentrations are dose-normalized to 1 mg/kg. The missing 24 h time point after intravenous dosing is due to absent sampling in CBD and due to all concentrations being <LLOQ in CBC.

**Table 1:** Mean ± SD Serum Pharmacokinetic Parameters of CBD, CBG, CBC, and CBN after Administration in the Form of an Oil Solution (oral) and Nanoemulsion (Oral and Intravenous) to Rats^a^

	CBD			CBG			CBC			CBN		
	Oil	Emulsion		Oil	Emulsion		Oil	Emulsion		Oil	Emulsion	
	PO	PO	IV	PO	PO	IV	PO	PO	IV	PO	PO	IV
N	6	7	7	8	5	6	8	6	6	8	7	7
C_max_ (ng/mL)	27 ± 10*	39 ± 8	845 ± 253	15 ± 15	6 ± 2	1186 ± 286	2.6 ± 1.5*	12 ± 2	935 ± 147	15.3 ± 4.6	12 ± 3	1226 ± 252
T_max_ (h)	6.8 ± 1.9*	3.2 ± 0.9	-	4.9 ± 2.0*	2.0 ± 1.3	-	6.0 ± 1.3*	3.6 ± 1.9	-	6.5 ± 1.2*	2.2 ± 0.6	-
T_1/2_ (h)	4.8 ± 0.8	4.4 ± 0.8	2.5 ± 0.5	3.1 ± 1.3	5.1 ± 4.1	2.5 ± 1.6	13 ± 19	5.8 ± 1.9	2.2 ± 0.4	4.2 ± 1.7	3.5 ± 1.1	5.3 ± 1.8
AUC_0‑inf_ (ng h/mL)	205 ± 57	256 ± 38	623 ± 141	36 ± 22	28 ± 10	716 ± 136	31 ± 23*	117 ± 33	640 ± 85	148 ± 57*	91 ± 19	1041 ± 212
F_0‑inf_ (%)	52 ± 19	45 ± 18	-	12 ± 11	4.4 ± 2.6	-	34 ± 18	18 ± 5	-	20 ± 7*	9.2 ± 2.6	-
V_ss_ (L/kg)	-	-	2.9 ± 1.1	-	-	7.3 ± 2.3	-	-	11 ± 2.2	-	-	6.5 ± 1.6
CL (L/h kg)	-	-	1.7 ± 0.6	-	-	5.9 ± 1.1	-	-	6.0 ± 0.7	-	-	3.0 ± 0.7

a The administered dose was 10 mg of CBD and 15 mg of the other cannabinoids in oral dosing (volume 1 mL). The intravenous doses were 1 mg of CBD and 1.5 mg of the other cannabinoids (volume 0.1 mL). All concentrations and AUCs are dose-normalized to 1 mg/kg. CBD intravenous data have already been published earlier.^28^ * marks a significant difference (*p* < 0.05) between nanoemulsion and the corresponding oil solution.

The absolute oral bioavailability of 52% and 45% in CBD oil solution and nanoemulsion, respectively, (Table 1) exceeds significantly reports published so far (8.5% for a lipid-free formulation,^19^ 14% for sunflower oil,^28^ 22% for a long chain triglyceride-based formulation^19^ and 25% for a sunflower oil-based microemulsion).^28^ The reason may lie in the extended time over which the pharmacokinetic profile was measured in this study (24 h and extrapolated to infinity). The older studies usually used a shorter period of measurement which may not have covered the full pharmacokinetic profile and a significant portion of AUC was not captured.

The absolute oral bioavailability was generally very different between the cannabinoids (Oil solution vs nanoemulsion: 52% vs 45% for CBD, 12% vs 4% for CBG, 34% vs 18% for CBC, and 20% vs 9% for CBN) despite their similar chemical properties and despite administration in the same formulations. This indicates that the factors affecting the amount of systemically available drug (intestinal absorption, intestinal and liver first-pass metabolism, extent of lymphatic transport) have to play varying roles in the particular compounds. In the lymphatic transport studies, the extent of portally absorbed drug (F_AP_) in the nanoemulsions did not differ significantly between the cannabinoids, whereas the relative proportion of lymphatically absorbed drug (F_RL_) was rather low in CBD and CBG (both 13%) and higher in CBC and CBN (47% and 36%, respectively), see below. The situation was different for the oil solutions, where the F_AP_ in CBG was lower compared to other cannabinoids, but the relative bioavailability via lymph was very high (>50%) similarly in all four compounds.

### 3.2 Lymphatic Transport

Figure 3 displays CBD, CBG, CBC and CBN serum and lymph profiles and the corresponding cumulative lymphatic transport after administration to anesthetized lymph duct-cannulated rats. Table 2 summarizes all the lymphatic transport parameters. Administration of a nanoemulsion led generally to an increased total absolute bioavailability compared to oil solution. The bioavailability increase was due to both increased direct absorption into portal blood and transport through lymphatic vessels with the exception of CBD, where the lymphatic transport measured as absolute bioavailability via lymph (F_AL_) did not change significantly. Interestingly, the absolute bioavailability via portal vein increased more than the bioavailability via lymph in all four cannabinoids. Whereas there was no increase, 8-fold, 3-fold, and 3-fold increase in F_AL_ for CBD, CBG, CBC and CBN, respectively, there was an 11-fold, 71-fold, 8-fold and 13-fold increase in absolute bioavailability via portal vein (F_AP_) (Table 2). Consequently, the relative bioavailability via lymph (F_RL_) dropped by 74% in CBD, 75% in CBG and 48% in CBN in the nanoemulsion compared to oil solution. The F_RL_ decrease in CBC did not reach statistical significance (p = 0.12). The reason for the higher bioavailability via portal vein (F_AP_) in all cannabinoids and higher bioavailability via lymph (F_AL_) in all cannabinoids but CBD for the nanoemulsion compared to oil solution lies probably in a poor dispersion and digestion of oil that was administered intraduodenally, skipping the dispersion and digestion process in the stomach.^3^

**Figure 3:**
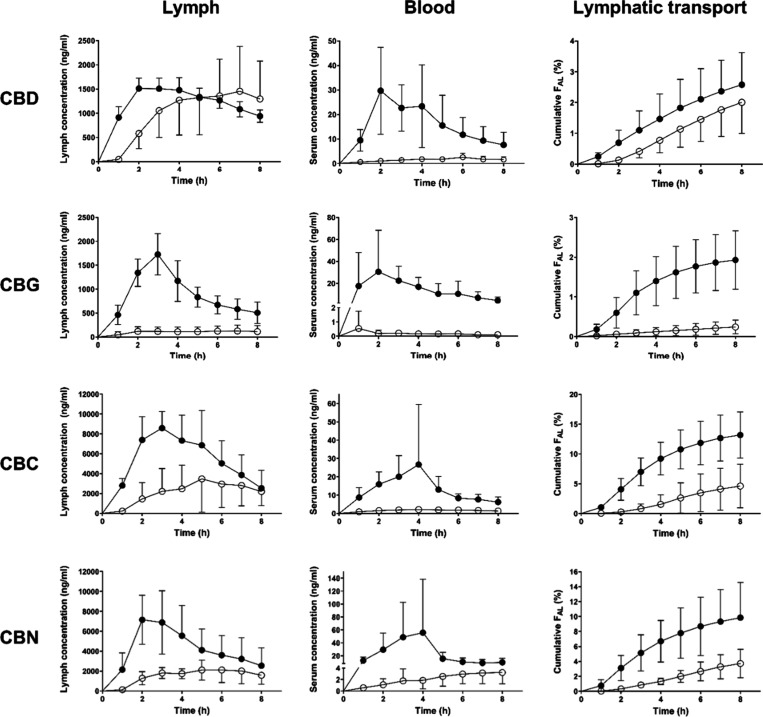
Mean ± SD serum and lymph profiles and cumulative lymphatic transport of CBD, CBG, CBC, and CBN after intraduodenal administration to anesthetized lymph duct-cannulated rats in a form of nanoemulsion (full circles) and oil solution (empty circles), *n* = 6 or 7; see Table 2. The administered dose was 10 mg of CBD and 15 mg of the other cannabinoids. The administered volume was 1 mL. All concentrations are dose-normalized to 1 mg/kg. F_AL_, absolute bioavailability via lymph.

**Table 2:** Mean ± SD Lymphatic Transport Parameters of CBD, CBG, CBC, and CBN after Intraduodenal Administration to Anesthetized Lymph Duct-Cannulated Rats in a Form of Nanoemulsion and Oil Solution^a^

	CBD		CBG		CBC		CBN	
Formulation	Emulsion	Oil	Emulsion	Oil	Emulsion	Oil	Emulsion	Oil
N	6	6	7	6	7	6	6	7
AUC_0–8_ lymph (ng h/L)	9556 ± 996	7699 ± 3815	7026 ± 1159*	802 ± 518	43,020 ± 11,165*	16,695 ± 13,034	33,935 ± 12,019*	11,979 ± 4,006
AUC_0–8_ serum (ng h/L)	125 ± 54*	11 ± 5	117 ± 71*	1.6 ± 1.7	98 ± 46*	12 ± 4	187 ± 93*	16 ± 8
Lymph/serum AUC ratio	100 ± 60*	699 ± 333	77 ± 31*	802 ± 582	530 ± 239*	1255 ± 676	274 ± 231*	976 ± 519
F (%)	24 ± 9*	4.0 ± 1.5	19 ± 10*	0.5 ± 0.3	29 ± 7*	6.6 ± 3.9	29 ± 10*	5.3 ± 2.0
F_AP_ (%)	21 ± 9*	1.9 ± 0.9	17 ± 10*	0.24 ± 0.25	16 ± 8*	2.0 ± 0.7	20 ± 10*	1.6 ± 0.9
F_AL_ (%)	2.6 ± 1.0	2.0 ± 0.9	1.9 ± 0.7*	0.24 ± 0.16	13 ± 4*	4.6 ± 3.3	9.9 ± 4.3*	3.7 ± 1.8
F_RL_ (%)	13 ± 8*	50 ± 12	13 ± 7*	51 ± 24	47 ± 14	63 ± 17	36 ± 20*	69 ± 13

a The administered dose was 10 mg of CBD and 15 mg of the other cannabinoids. The administered volume was 1 mL. All AUCs are dose-normalized to 1 mg/kg. * marks a significant difference (*p* < 0.05) between nanoemulsion and the corresponding oil solution. F, total absolute bioavailability; F_AP_, absolute bioavailability via portal vein; F_AL_, absolute bioavailability via lymph; F_RL_, relative bioavailability via lymph.

As expected, the lymphatic transport played a major role in the general pharmacokinetics of all four cannabinoids tested. The relative bioavailability via lymph (F_RL_) of ≥50% seen after administration of oil solutions counts the cannabinoids among compounds with the highest lymphotropic potential, like vitamin D or halofantrine.^10,35^

### 3.3 Log P Measurement

Due to the highly lipophilic character of cannabinoids, their concentration in the organic phase is several orders of magnitude higher than the concentration in the aqueous phase. As a result, the difference in the total amount of cannabinoids and the amount present in the organic phase is very small and falls within the experimental error of the analytical method. Therefore, log P values were calculated based on the measured cannabinoid concentration in the aqueous phase, while the concentration in the octanol phase was calculated from the initial amount of the cannabinoid used and its measured concentration in the aqueous phase. Using this approach, log P values were estimated as 6.2 ± 0.2 for CBN and 7.2 ± 0.2 for CBG. Due to the very low concentrations of CBD and CBC in the aqueous phase, which fell below the LOQ of both methods, we were unable to determine the log P for these two cannabinoids. Nevertheless, based on the LOQ values, we can infer that the log P values exceed 6.3 for CBD and 6.7 for CBC.

It is clear that molecules with log P > 5 can be transported through the mesenteric lymph while molecules with log P < 5 cannot.^2^ However, even in molecules with log P > 5, the F_RL_ can still profoundly vary. Reason for a very low F_RL_ (<10%) can be an insufficient amount of long-chain triglycerides present in the drug formulation to stimulate the chylomicron assembly like it was the case in halofantrine (log P 8.9) when administered in an aqueous solution (F_RL_ = 5.3%).^10^ A very high F_RL_ (>90%), on the other hand, is usually seen in drugs with a prominent first-pass metabolism in the liver, like testosterone undecanoate (log P 6.7, F_RL_ = 98.5%).^6^ This high F_RL_ does not mean that no drug is absorbed into the portal vein and all the drug is absorbed into the lymphatics, but rather that the drug absorbed into the portal vein is (almost) completely metabolized during the first pass in the liver and only lymphatically transported portion reaches the systemic circulation. The mechanisms determining the F_RL_ in the range ∼10–90% are not well understood. Important here is the type and amount of lipids administered. Long-chain triglycerides stimulate the lymphatic transport more than medium-chain triglycerides and short-chain triglycerides. The F_RL_ of halofantrine was 70%, 29% and 15% when administered in a long, medium and short-chain triglyceride oil, respectively.^10^ In this study, cannabinoids administered in a sunflower oil, which consists mainly of long chain triglycerides containing oleic and linoleic acid, reached a very high F_RL_ (50–69%). In the nanoemulsion, on the other hand, there were no standard triglycerides. It consisted of polyethoxylated castor oil (Kolliphor EL), diethylene glycol monoethyl ether, and propylene glycol monocaprylate. The lack of long chain triglycerides is the most plausible explanation, why the F_RL_ here was lower (13–47%).

### 3.4 CBD, CBG, CBC, and CBN Quantitative Structure–Activity Relationship with Lymphatic Transport

Table 3 summarizes the basic physicochemical properties and quantitative structural parameters of the four cannabinoids and Figure 4 shows their relation to lymphatic transport expressed as mean relative bioavailability via lymph (F_RL_). There was a highly significant negative correlation between the smallest orthogonal molecular dimension and F_RL_ marked by the significantly nonzero slope in the linear regression analysis (*p* < 0.001). Topological polar surface area did also negatively correlate (p = 0.04). Other physicochemical properties (medium and largest orthogonal dimension, molecular volume, rotatable bond count, log P and p*K*_a_) did not have a significant effect.

**Table 3:** Physicochemical Properties and Quantitative Structure Parameters of the Four Tested Cannabinoids

	CBD	CBG	CBC	CBN
Log P	>6.3	7.2	>6.7	6.2
p*K*_a_	9.70	9.71	9.68	9.40
Rotatable bond count	6	9	7	4
Topological polar surface area (Å^2^)	40.5	40.5	29.5	29.5
Molecular volume (Å^3^)	329	339	329	312
Largest orthogonal dimension (Å)	17.2	15.3	16.4	17.1
Medium orthogonal dimension (Å)	9.9	10.5	11.7	10.6
Smallest orthogonal dimension (Å)	8.31	8.12	7.10	6.58
Medium/largest orthogonal dimension ratio	0.576	0.687	0.713	0.619
Smallest/largest orthogonal dimension ratio	0.483	0.530	0.433	0.386

**Figure 4:**
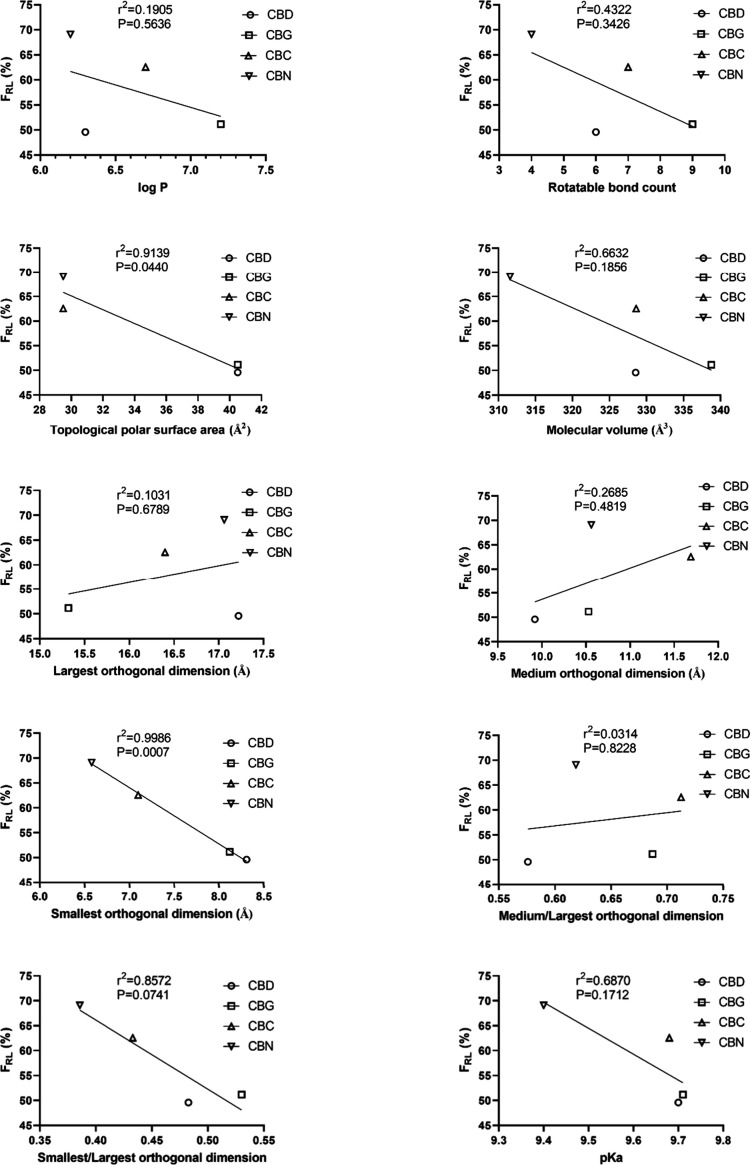
Relation between mean relative bioavailability via lymph of CBD, CBG, CBC, and CBN and their physicochemical properties and quantitative structure parameters after administration in an oil solution. The log P values for CBD and CBC shown in the graph are the minimal values estimated by the measurement which are very close to the calculated cLogP P values (6.3< vs 6.5 in CBD and 6.7< vs 6.9 in CBC). F_RL_, relative bioavailability via lymph.

In our study, we compared F_RL_ of four highly lipophilic cannabinoids administered in the very same formulation. Interestingly the F_RL_ did not correlate with the log P value. Based on these results and based on the literature, it seems that log P of 5 represents a threshold beyond which a drug can be lymphatically absorbed but other factors determine to which extent. Our QSAR analysis on a limited number of compounds showed that the overall size of the molecule seems not to be so important for increasing lymphatic uptake as its planarity (F_RL_ increases with decreasing smallest orthogonal dimension, while the largest and medium orthogonal dimensions and volume of the molecule are not significantly associated with F_RL_). Another structural parameter that (negatively) correlates with the F_RL_ is the topological polar surface area.

Following oral administration, strongly lipophilic drugs access the mesenteric lymph vessels via absorption across enterocytes.^4^ The substances are drawn into the cell organelles, especially the smooth endoplasmic reticulum, where the formation of chylomicrons takes place. The chylomicrons consist of a phospholipid membrane, proteins and a lipophilic core consisting mainly of triglycerides, in which the highly lipophilic substances, including drugs, are dissolved. Chylomicrons are subsequently transported by the Golgi apparatus toward the basal pole of the cell and secreted into the intercellular space. They are too large to penetrate the wall of blood capillaries and, therefore, enter the lymphatic capillaries, which have a more permeable vessel wall. Thus, the interpretation of our observations may be that higher planarity allows easier diffusion across phospholipid membranes of the cell organelles where the chylomicrons are formed and across the membranes of the chylomicrons themselves. The lower topological polar surface area leads additionally to higher drug solubility in long-chain triglycerides in the chylomicron lipophilic cores. To our knowledge, this is the first report describing these structure–activity relationships that are important for the design and development of modern intestinal lymph-targeting drugs. Further studies are warranted to confirm these preliminary conclusions.

## 4 Conclusions

This work shows on the example of four cannabinoids CBD, CBG, CBC, and CBN that for highly lipophilic active compounds, planarity of the molecule and less surface polarity seem to be crucial structural features that facilitate lymphatic transport and could therefore play an important role in this absorption mechanism in the context of absolute oral bioavailability of the drug. The overall size of the molecule was of little significance for the extent of lymphatic absorption. Compared to standard oil solution, administration of a nanoemulsion devoid of standard triglycerides increased the absorption speed and amount of cannabinoids absorbed directly into portal blood, bud did not affect the overall bioavailability. Overall, intestinal lymphatic transport plays a major role in the process of intestinal absorption of the cannabinoids CBD, CBG, CBC, and CBN.

## Supplementary Material



## Notes

Thorough information on the nanoemulsion formulation development and optimization (PDF)
