# Supplementary material for: Effect of Quantitative Structural Properties and Drug Formulation in Four Cannabinoids (Cannabidiol, Cannabigerol, Cannabichromene, and Cannabinol) on Their Lymphatic Transport after Enteral Administration in Rats
Source: Mol Pharm. 2025 Jul 4;22(8):4544–55. doi: 10.1021/acs.molpharmaceut.4c01357 (PMC12326358; doi:10.1021/acs.molpharmaceut.4c01357)
Supplement: Supplementary file 1 [file mp4c01357_si_001.pdf]

Effect of quantitative structural properties and drug formulation in four cannabinoids (cannabidiol, cannabigerol, cannabichromene and cannabinol) on their lymphatic transport after enteral administration in rats

Pavel Ryšánek[a], Petr Jelínek[b], Hynek Housar[b], Petr Kozlík[c], Tomáš Křížek[c], Anežka Nováková[a], Michaela Sklenárová[a], Viktória Paulusová[a], Sara Merdita[a], Mahak Arora[a], Olesia Symkanych[a], Monika Šteigerová[a], Eliška Zmeškalová[b, d], Ondřej Slanař[a]*, Miroslav Šoóš[b], Martin Šíma[a]

[a]Institute of Pharmacology, First Faculty of Medicine and General University Hospital in Prague, Charles University, Albertov 4, 128 00 Prague, Czech Republic

[b]Department of Chemical Engineering, Faculty of Chemical Engineering, University of Chemistry and Technology, Technická 3, 166 28 Prague, Czech Republic

[c]Department of Analytical Chemistry, Faculty of Science, Charles University, Hlavova 2030/8, 128 43, Prague, Czech Republic

[d]Institute of Physics of the Czech Academy of Sciences, Na Slovance 2, 182 00 Prague, Czech Republic

***Corresponding author:**
Ondřej Slanař
Institute of Pharmacology, First Faculty of Medicine and General University Hospital in Prague, Charles University, Albertov 4, 128 00 Prague, Czech Republic

Tel.: +420224968146
ondrej.slanar@lf1.cuni.cz

Supplementary Material

Nanoemulsion Formulation

To test pharmacokinetic parameters for various cannabinoids, the self-emulsifying drug delivery system (SEDDS) was selected due to its facile preparation and as it has shown an enhanced absorption profile for CBD.[1] However, prior to administration the oil phase was mixed with water forming nanoemulsion to decrease viscosity and facilitate application to rats. Hence, the formulation is in the paper referred to as nanoemulsion.

In general, the nanoemulsion was prepared as follows. First, the oil phase containing Propylene glycol monocaprylate (PGMC), surfactant Kolliphor EL (CR-EL), co-surfactant and cannabinoid was prepared and thoroughly homogenized. As co-surfactants, we tested three compounds: Diethylene glycol monoethyl ether (TRSC) or Ethyl alcohol (ETOH) or Propylene glycol (PRGL). Next, four parts of water with respect to one part of oil (by weight) were added dropwise to the oil mixture at mild stirring. The stability of prepared nanoemulsions (initially without cannabinoids) was evaluated using dynamic light scattering (LS Instruments, Switzerland). Microemulsions and unstable samples, characterized by very high turbidity or phase separation, respectively, were not further analyzed. Optimization results are shown in the form of ternary diagrams for three tested co-surfactants (TRSC, ETOH, PRGL) in **Fig. S1**.

Optimization Results

According to the manufacturer, CBD is most soluble in PGMC among other products.[2] High solubility was also expected for other cannabinoids, thus PGMC was chosen as a suitable oil-phase base for nanoemulsion formulation in this study. Next, CR-EL was selected as a surfactant capable of forming nanoemulsions with PGMC in a wide range of concentrations (based on previous non-published experiments). To optimize the composition, stability and the size dependence of various emulsion mixtures were investigated for systems containing the aforementioned components and selected co-surfactants – TRSC, ETOH and PRGL (without cannabinoids). According to the optimization study, TRSC showed the best performance in the formation of stable nanoemulsions with desirable sizes (**Fig. S1**). Furthermore, TRSC is a proven skin permeation enhancer and solubilizer. Moreover, as shown by Franceschinis et al., TRSC acted as an excellent absorption

enhancer for 4,6,4′trimethylangelicin during oral administration.[3, 4] Hence, TRSC was chosen as a co-surfactant and solubilizer for the final formulation tested in the pharmacokinetic study.

The final composition of nanoemulsion was chosen based on the compromise between the cannabinoids' loading capacity and the used surfactant amount. The final formulation contained 50wt.% of CR-EL, 30wt.% of TRSC and 20wt.% of PGMC. Long-term stability for the optimized formulation was tested for two cannabinoid concentrations – 15 mg/ml and 30 mg/ml. Results are shown in **Tab. S1**. The size was below 30 nm even after 30 days for all cannabinoids at 15mg/ml concentration. A variable size decrease was observed for higher concentration, however, the size is comparable after 30 days except for CBG, for which the formed nanoemulsion was not stable. Although all formulations were stable over time and to avoid any colloid instability and cannabinoid decomposition, the water phase was added to the oil phase directly before application in pharmacokinetic study.

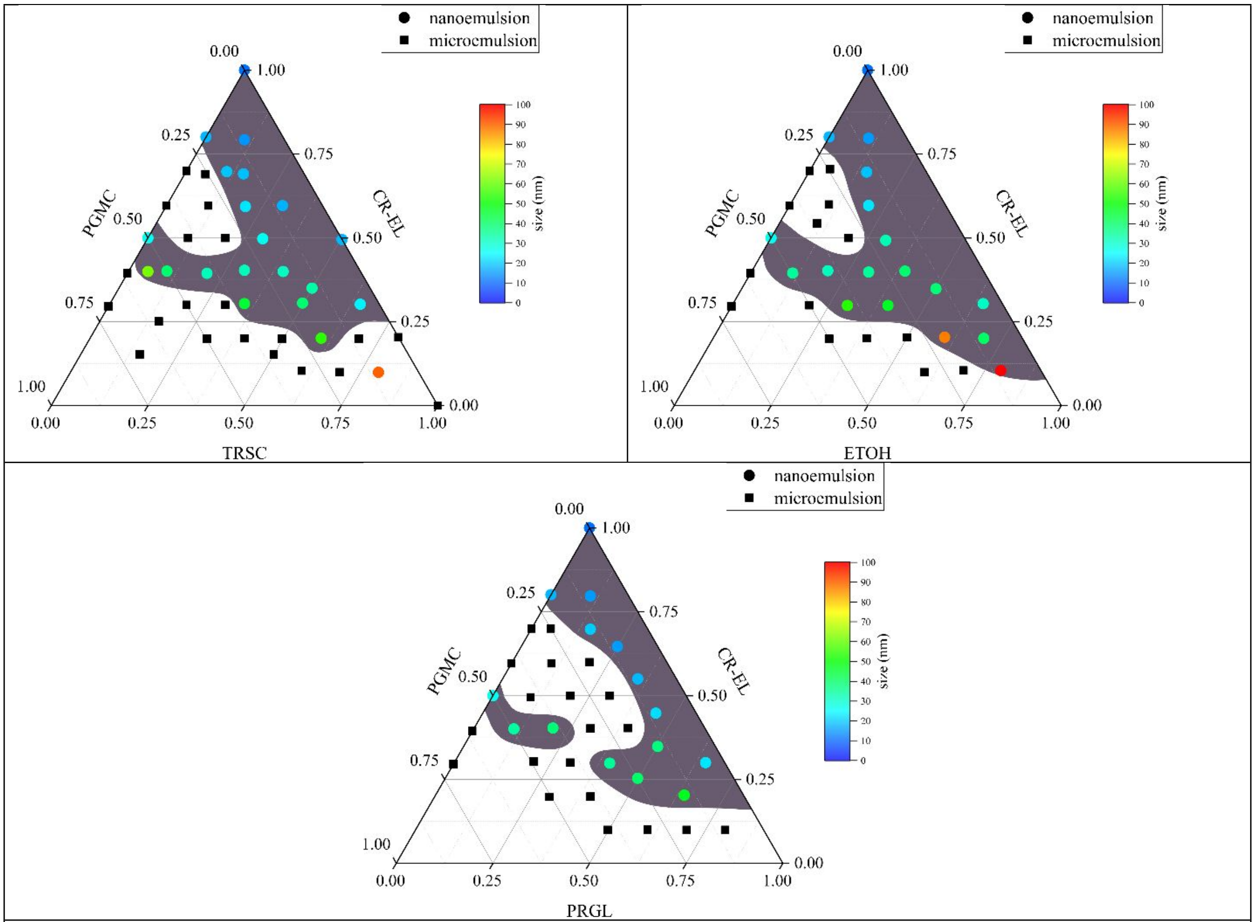

Fig. S1. Ternary diagrams of nanoemulsion for tested co-surfactants

Tab S1. Colloidal stability of the final formulation for tested cannabinoids

DLS, 90° [nm]	15 mg/ml		30 mg/ml	
	1 day	30 days	1 day	30 days
CBD	26	26	200	222
CBG	31	32	-	-
CBC	25	25	40	34
CBN	27	27	67	68

1. De Pra, M. A. A.; Vardanega, R.; Loss, C. G. Lipid-based formulations to increase cannabidiol bioavailability: In vitro digestion tests, pre-clinical assessment and clinical trial. *Int J Pharm* **2021,** *609*, 121159.
2. Gattefossé. Formulating cannabinoids with lipid excipients. **2020**.

3. Osborne, D. W.; Musakhanian, J. Skin Penetration and Permeation Properties of Transcutol(R)-Neat or Diluted Mixtures. *AAPS PharmSciTech* **2018,** *19*, (8), 3512-3533.
4. Ha, E. S.; Lee, S. K.; Choi, D.; Jeong, S. H.; Hwang, S. J.; Kim, M. S. Application of diethylene glycol monoethyl ether in solubilization of poorly water-soluble drugs. *J Pharm Invest* **2020,** *50*, (3), 231-250.
